# Household Mealtimes During the 2020 COVID-19 Lockdown in Aotearoa New Zealand: The Influence of Household Type and Psychological Distress

**DOI:** 10.3389/fnut.2022.855866

**Published:** 2022-06-14

**Authors:** Victoria Egli, Lauren Hunter, Rajshri Roy, Lisa Te Morenga, Charlotte De Backer, Lauranna Teunissen, Isabelle Cuykx, Paulien Decorte, Sarah Gerritsen

**Affiliations:** ^1^School of Nursing, Faculty of Medical and Health Sciences, University of Auckland, Auckland, New Zealand; ^2^School of Population Health, Faculty of Medical and Health Sciences, University of Auckland, Auckland, New Zealand; ^3^Faculty of Medical and Health Sciences, University of Auckland, Auckland, New Zealand; ^4^Massey University, Wellington, New Zealand; ^5^Faculty of Social Sciences, University of Antwerp, Antwerp, Belgium

**Keywords:** COVID-19, diet, mealtime, eating behavior, family, household, nutrition

## Abstract

COVID-19 lockdown meant disruptions to daily routines for households in Aotearoa New Zealand. The research presented here investigates how mealtimes changed for people living in New Zealand during the first COVID-19 lockdown in mid-2020 and sought to determine if household composition type and psychological distress impacted the frequency of engaging in several mealtime behaviors. The COVID Kai Survey collected data using an anonymous, online survey and asked questions on sociodemographic characteristics including household composition, frequency of engaging in different mealtime behaviors before and during lockdown, and psychological distress, which was measured using the Kessler 6 screening tool. The findings of this study shows an increase in the perceived importance of mealtimes (*n* = 807, 26.9% before lockdown, *n* = 1,154, 38.5% during lockdown) and an increase in the proportion of the survey respondents who stated that they frequently ate meals at the dinner table (*n* = 1,343, 44.8% before lockdown, *n* = 1,481, 49.4% during lockdown). There was a decrease, across all household composition types, in the proportion of respondents who ate out frequently at a restaurant or café (*n* = 878, 29.3% before lockdown, *n* = 5, 0.2% during lockdown, P < 0.001). The use of meal kits, e-dining, and eating meals in front of screens is also presented and discussed. All results are discussed with reference to Aotearoa New Zealand’s stringent lockdown restrictions. Respondents who experienced psychological distress during lockdown were 1.47 times more likely to consider mealtimes an important part of their day and respondents living in households with one adult and at least one child who also experienced psychological distress were 5.95 times more likely to eat dinner at the dinner table than those who did not report psychological distress. Findings of this study further the understanding of the wider societal impact of COVID-19 lockdown on everyday life.

## Introduction

Aotearoa New Zealand had one of the most effective responses to COVID-19 worldwide, eliminating the virus for large parts of 2020 and 2021 (1, 2). To achieve this success, Aotearoa New Zealand had a particularly stringent lockdown during the period of the 25th of March to the 13th of May 2020 (3). Overnight, people had to limit their movements, schools shut, restaurants and retail stores closed with only supermarkets and pharmacies remaining open for essential food and medical supplies, and employees were instructed to work from home wherever possible. Consequently, people’s habits and daily routines suddenly changed.

The COVID-19 crisis resulted in many changes to the way people prepared and consumed food and the variety of food they could access. During lockdown, New Zealanders had to cook for themselves, not meet up with others for social and culturally important meals, and contend with panic-buying (4), empty shelves at supermarkets and fear of infection. On top of spending more time at home, people had to deal with the uncertainty and additional stress that accompanies the current COVID-19 pandemic.

There is a growing evidence base to show that our eating behaviors change when we are stressed (5). Specific to the COVID-19 lockdown, many have experienced additional stress due to isolation and fear of infection, disruption to food supply chains, increased food insecurity, potential job losses and financial hardship (6). Previous coping mechanisms for times of stress and hardship such as sharing meals (4) may have been prohibited for some during lockdown. In response e-dining, the practice of engaging in a meal with other people electronically through Zoom, Facetime, or other video chatting software, emerged to help people feel connected to one another but without the obvious sharing of food (7).

Household composition is an important consideration of how society responds to crisis like COVID-19 because of the association between household composition and primary drivers of stress, particularly financial hardship and stress related to responsibilities such as caring for children and aging parents (8, 9). The groups most vulnerable to increasing food insecurity after a crisis are women, ethnic minorities, immigrants, single-parent households, and low-income families (10, 11). Household composition in New Zealand takes on a myriad of forms from single person households, to households with many adults and no children, to households with 1 or more adults and 1 or more children (12). The effectiveness of many of New Zealand’s public health measures to control COVID-19 were centered on the household, specifically the ability to isolate and maintain physical distancing (13). Globally additional challenges, including purchasing and safely preparing healthy food, were faced by those living in overcrowded households or unhealthy housing during lockdown (14).

For many people, food is a way to mitigate or manage stress. Globally, people who reported less stress in lockdown had healthier overall eating behaviors and made healthier food choices than people who reported high levels of stress (15, 16). The practice of emotional eating and increased consumption of sugary, salty, and fatty foods are associated with increased stress levels during lockdown (17). In times of uncertainty, mealtimes can help people maintain a sense of normalcy and feeling connected to other people in their household (18). More frequent family mealtimes and more pleasant mealtime atmospheres are associated with a variety of positive health and wellbeing outcomes including better nutrition, higher social competence, and fewer emotional and behavioral problems (19, 20). Daily routines and the structure associated with regular mealtimes can help people manage stress and maintain healthy habits (20–22). Mealtime behaviors include things such as where an individual eats their meals (at the dinner table vs. in front of the television vs. at a restaurant), who they eat with (eating alone vs. eating with others), and where they acquire their meals (takeaway vs. cooking at home). However, there are many complexities associated with maintaining mealtime behaviors, such as time, disrupted food systems, lost income, and balancing other’s needs, and caring responsibilities (11, 22, 23).

This research aimed to investigate how mealtimes changed for New Zealanders during the first COVID-19 lockdown in mid-2020, and determine if household composition type and psychological distress impacted the frequency of engaging in several mealtime behaviors.

## Materials and Methods

This study reports on the findings of the COVID Kai Survey, the New Zealand arm of the international Corona Cooking Survey developed by researchers in Antwerp, Belgium. The Corona Cooking Survey was conducted in 38 countries, and over 37,000 people participated in the survey worldwide, with the results of the international study presented in De Backer et al. (23). The survey was uploaded onto the Qualtrics survey platform for each participating country to run independently. The questionnaire included questions regarding grocery shopping habits, food stockpiling habits, food preparation, cooking habits, ready-made meals vs. fresh/from scratch, self-perceived cooking ability, barriers to cooking and baking, decision making regarding recipe choice, self-perceived top food-related influential figures/organizations/brands, a food frequency questionnaire, source of nutrition advice, eating behaviors, perceived importance of mealtimes, lockdown conditions, psychological distress, and questions concerning sociodemographic characteristics (23).

The Corona Cooking Survey was granted ethical approval by the Ethics Advisory Committee on Social and Human Science at the University of Antwerp on April 16th 2020 (ref: SHW_20_46).

### The COVID Kai Survey

The Aotearoa New Zealand arm of the Corona Cooking Survey was called The COVID Kai Survey. It used exactly the same questions as the international version, only the invitation and introduction text were adapted to be appropriate to the population and culture of Aotearoa New Zealand. This was achieved by including Te Reo Mâori in the title, plus Statistics New Zealand’s standard ethnicity question was added to the questionnaire for ethnic group comparisons (24).

The COVID Kai Survey was released online in Aotearoa New Zealand on 24 April 2020 and remained open until 13 May 2020 (20 days total). During this time, Aotearoa New Zealand was under government-mandated Alert Level 3 and 4 restrictions. During Alert Level 4 restrictions in Aotearoa New Zealand, people were instructed to stay at home except for essential personal movement, and all businesses (except essential services) were closed. Grocery stores and pharmacies were open, but takeaway shops, restaurants and many small specialty food stores could not operate. During Alert Level 3 restrictions, schools remained closed, people were still instructed to stay within their household, some businesses could open with public health restrictions. Restaurants could open for contactless takeaway and delivery but could not open for dine-in meals.

The Aotearoa New Zealand arm of the study was granted ethical approval by the University of Auckland Human Respondents Ethics Committee on 24 April 2020 for 3 years (ref: 024607).

### Recruitment

Recruitment for the survey was through convenience and snowball sampling and was promoted widely through social media. Stakeholders, public food figures and colleagues from related organizations disseminated the survey invitation amongst their networks, and the general public shared the survey’s social media posts. Respondents were required to be aged 18 years or older and currently reside in New Zealand. Researchers monitored responses from demographic groups of interest multiple times during the data collection period. Facebook advertising was used to recruit groups with lower response rates, such as men and those aged over 65. After the data collection period closed, a NZ$3200 donation was given to The Foodbank Project (the Salvation Army) as koha (gift of gratitude) of $1 for each near-completed survey (24).

### Aim and Objectives

This study sought to investigate how mealtimes changed for New Zealanders during the first COVID-19 lockdown in mid-2020.

In the objectives below mealtime behaviors refers to: the perceived importance of mealtimes, the frequency of eating at the dinner table, frequency of watching television or another screen while eating a meal, frequency of engaging in e-dining and use of meal service kits.

Objective 1: To determine if household composition type impacted the frequency of engaging in several mealtime behaviors during the first COVID-19 lockdown in mid-2020.

Objective 2: To determine if psychological distress impacted the frequency of engaging in several mealtime behaviors during the first COVID-19 lockdown in mid-2020.

Objective 3: To determine if there is an association between household composition type and psychological distress experienced by participants during the first COVID-19 lockdown in 2020.

### Data Preparation

The COVID Kai Survey closed with *n* = 3,574 entries. *n* = 574 responses were removed from the final dataset due to implausible answers or not answering all relevant questions specifically: mealtime behaviors (*n* = 568), use of meal services (*n* = 2), and frequency of e-dining (*n* = 4). One respondent was removed as their stated age of 120 years was deemed implausible and so the accuracy of the rest of their responses was questionable. *n* = 3,000 responses are included in the analyses presented.

### Variables

The original COVID Kai Survey contained 100 variables, including questions regarding perceived cooking ability, a food frequency questionnaire, and sources of nutritional advice, amongst other topics. Many of these variables have been discussed elsewhere (24–26).

The sociodemographic information collected included age, gender, ethnicity, highest education qualification, employment status before and during lockdown, financial struggle before and during lockdown, and whether respondents lost any income during lockdown. Respondents also shared the number of children and/or adults they were currently living with. These data were used to create the following household composition subgroups: single person households, households with 2 + adults and no children, households with 2 + adults and 1 + child, households with 1 adult and 1 + child. Age group categories were also created (18–29, 30–49, 50–69, 70 +) and ethnicity was coded following the guidelines published by the Ministry of Health, Health Information Standards Organization (27). When respondents included multiple ethnicities, the ethnic groups were prioritized according to Statistics New Zealand prioritization categories and only coded once, in line with common practice in Aotearoa New Zealand (28). The ethnic categories included in this analysis were “Māori,” “Pacific,” “Asian,” and “New Zealand European/Other (NZEO).”

Psychological distress was measured using the questions from the Kessler-6 test (29). This six-item inventory uses a Likert scale to identify the level of psychological distress an individual is currently experiencing. The Kessler-6 test asks respondents to self-report how they have been feeling over the past 2 weeks; however, respondents were asked to answer the questions during the lockdown period for this survey, which was between 32 and 52 days, while the survey was open. The original Kessler-6 test is conducted using a 5-point Likert scale; the possible response options are “never,” “a little of the time,” “some of the time,” “most of the time,” and “all of the time.” The data collected in the COVID Kai Survey was collected on a 7-point Likert scale; the possible responses were “never,” “very rarely,” “rarely,” “sometimes,” “frequently,” “very frequently,” and “all the time.” To address this discrepancy, the responses from the 7-point scale were adjusted to best fit the 5-point scale used in the original Kessler-6 tool so that the same cut-point of 13 or greater could be used as the indicator of psychological distress (30). The responses “very rarely” and “rarely” were combined to become “a little of the time,” and the responses “frequently” and “very frequently” were combined to become “most of the time.”

The questions regarding mealtime behaviors included: asking respondents to rate how important mealtimes were for them and their household, how frequently respondents ate dinner at their dinner table, how frequently respondents watched television or another screen while eating a meal, and how frequently respondents engaged in e-dining. These questions had a 7-point frequency response scale ranging from “never” to “all the time.” Respondents were asked to report their behavior on the scale twice, once at the time of survey completion (during lockdown) and once before the COVID-19 lockdown began.

The questions regarding the use of meal services included: asking respondents how often they eat out in a café or restaurant, how often they use delivery or takeaway services, and how often they use meal or ingredient boxes. Respondents reported their behavior before and during the lockdown using a 7-point Likert scale.

All data was collected cross-sectionally, during the lockdown. For some of the mealtime behavior questions, respondents were asked to recall their behavior before the lockdown and report their behavior at the time of survey completion. In the analysis of all mealtime behavior variables, the data were presented as binary categories (frequently or less than frequently). Variables such as frequency of e-dining and psychological distress were only collected for one point in time (at the time of the survey during the lockdown) and therefore analyzed for differences between groups. The responses for frequency of e-dining were grouped into three categories;(almost) never, once a week or less, and more than once a week.

### Analysis

Three main types of analysis were conducted for this study: descriptive statistics, comparison of behaviors during lockdown to before lockdown, and differences in mealtime behaviors between household composition groups. All data were analyzed both for all survey respondents and broken down by household composition subgroups. Fisher exact and Wald tests were used to determine differences between household composition groups. Multivariate logistic regression, adjusted for demographic covariates, was run for four variables: perceived mealtime importance, frequency of eating at the dinner table, frequency of eating in front of a screen, and use of meal kit services. Covariates were decided *a priori* and included: household composition type, age group, gender, and ethnicity. The household group “households with 2 or more adults and 1 or more child” was chosen as the reference group for the regression as they had the largest sample size and likely had the best health outcomes.

The impact of psychological distress on mealtime behaviors was tested using logistic regression, predicting each behavior by psychological distress score.

All analyses were conducted in R Studio.

## Results

### Sociodemographic Characteristics

The majority of survey respondents identified as female (*n* = 2,658, 88.6%). 30–49-years accounted for nearly half (*n* = 1,429, 47.6%) of all survey respondents. The largest ethnic group of respondents was New Zealand Europeans or Other (*n* = 2,472, 82.4%). Māori made up *n* = 315, 10.5% of the respondents. Asian and Pacific people made up the remainder of the participant ethnic groups (*n* = 132, 4.4%, and *n* = 81, 2.7%, respectively).

Around half of the respondents (*n* = 1,586, 52.9%) worked full-time before lockdown. A quarter of survey respondents (*n* = 774, 25.8%) stated that they had lost some or all their income during lockdown. Those living in households of multiple adults with no children experienced the greatest rates of lost income (*n* = 434, 27.1%). Those living in households with one adult and at least one child experienced the lowest rate of income lost (*n* = 9, 18.8%). Most respondents reported that they rarely or never struggled financially during lockdown (*n* = 1,809, 60.3%) with a smaller proportion (*n* = 298, 9.9%) reporting to have struggled financially often or all of the time and almost a third (*n* = 811, 27%) reporting they struggled to buy food during lockdown often or all of the time. The highest level of financial struggle were households with one adult and at least one child (*n* = 13, 27.1%). Detailed sociodemographic characteristics of survey respondents by household composition are presented in Table 1.

**TABLE 1:** Sociodemographic characteristics.

Descriptive statistic, *n* (%)					

	Total sample	Single person households	Households with 2 + adults and no children	Households with 2 + adults and 1 + child	Households with 1 adult and 1 + child
	3,000 (100)	292 (100)	1,601 (100)	1,059 (100)	48 (100)
**Gender**					
Female	2,658 (88.6)	262 (89.7)	1,414 (88.3)	938 (88.6)	44 (91.7)
Male	311 (10.4)	28 (9.6)	165 (10.3)	114 (10.8)	4 (8.3)
Gender diverse	31 (1.0)	2 (0.7)	22 (1.4)	7 (0.7)	0 (0.0)
**Age group**					
18– < 30	508 (16.9)	18 (6.2)	405 (25.3)	83 (7.8)	2 (4.2)
30– < 50	1,429 (47.6)	95 (32.5)	504 (31.5)	795 (75.1)	35 (72.9)
50– < 70	948 (31.6)	145 (49.7)	613 (38.3)	179 (16.9)	11 (22.9)
70 +	115 (3.8)	34 (11.6)	79 (4.9)	2 (0.2)	0 (0.0)
**Ethnicity**					
NZEO	2,472 (82.4)	263 (90.1)	1,343 (83.9)	826 (78.0)	40 (83.3)
Māori	315 (10.5)	15 (5.1)	142 (8.9)	151 (14.3)	7 (14.6)
Pacific	81 (2.7)	6 (2.1)	33 (2.1)	41 (3.9)	1 (2.1)
Asian	132 (4.4)	8 (2.7)	83 (51.8)	41 (3.9)	0 (0.0)
**Employment status before lockdown**					
Not working	453 (15.1)	62 (21.2)	244 (15.2)	141 (13.3)	6 (12.5)
Student with or without job	227 (7.6)	12 (4.1)	159 (9.9)	55 (5.2)	1 (2.1)
Worked part-time	734 (24.5)	55 (18.8)	323 (20.2)	341 (32.2)	15 (31.3)
Worked full-time	1,586 (52.9)	163 (55.8)	875 (54.7)	522 (49.3)	26 (54.2)
**Employment status during lockdown**					
Not working	672 (22.4)	78 (26.7)	364 (22.7)	218 (20.6)	12 (25.0)
Student with or without job	220 (7.3)	13 (4.5)	149 (9.3)	56 (5.3)	2 (4.2)
Worked part-time	804 (26.8)	63 (21.6)	368 (23.0)	361 (34.1)	12 (25.0)
Worked full-time	1,304 (43.5)	138 (47.3)	720 (45.0)	424 (40.0)	22 (45.8)
**Income lost due to lockdown**					
Yes, at least some	774 (25.8)	66 (22.6)	434 (27.1)	265 (25.0)	9 (18.8)
No	2,226 (74.2)	226 (77.4)	1,167 (72.9)	794 (75.0)	39 (81.2)
**Struggled financially during lockdown**					
Often or all the time	298 (9.9)	38 (13.0)	123 (7.7)	120 (11.0)	13 (27.1)
Sometimes	893 (29.8)	89 (30.5)	456 (28.5)	321 (31.0)	19 (39.6)
Very rarely or never	1,809 (60.3)	165 (56.5)	1,021 (63.8)	604 (58.0)	16 (33.3)
**Struggled to buy food during lockdown**					
Often or all the time	811 (27.0)	81 (27.7)	394 (24.6)	314 (29.7)	22 (45.8)
Sometimes	283 (9.4)	31 (10.6)	144 (9.0)	99 (9.3)	9 (18.8)
Very rarely or never	1,906 (63.5)	180 (61.6)	1,063 (66.4)	646 (61.0)	17 (35.4)
					

### Mealtime Behaviors by Household Composition

There was an increase in the proportion of the survey respondents who stated that they frequently consider mealtimes to be an important part of their day during lockdown (*n* = 807, 26.9% before lockdown, *n* = 1,154, 38.5% during lockdown, *P* ≤ 0.001). This was a significant increase among all household composition groups except for households with one adult and at least one child (*P* = 0.823). These changes remained significant after adjusting for age, gender, and ethnicity differences (Table 2).

**TABLE 2:** Frequently*^a^* found mealtimes important before and during lockdown.

	Before lockdown, *n* (%)	During lockdown, *n* (%)	*p*-value^b^	Adjusted^c^ odds ratio (95%CI)	Odds ratio *p*-value^d^
Single person (*N* = 292)	53 (18.2)	86 (29.5)	0.002	1.9 (1.41, 2.55)	<0.001
Households with 2 + adults (*N* = 1,601)	437 (27.3)	600 (37.5)	<0.001	1.29 (1.08, 1.53)	0.005
Households with 2 + adults and 1 + child (*N* = 1,059)	304 (28.7)	453 (42.8)	<0.001	1.00 (REF)	–
Households with 1 adult and 1 + child (*N* = 48)	13 (27.1)	15 (31.3)	0.823	1.64 (0.88, 3.06)	0.120

*^a^Frequently classified as responses “Frequently,” “Very frequently,” or “All of the time.”*

*^b^Fisher’s exact test.*

*^c^Adjusted for age group, gender, and ethnicity. Ref = households with 2 + adults and 1 + child.*

*^d^Wald’s test.*

There was an increase in the proportion of the survey respondents who stated that they frequently ate meals at the dinner table during lockdown (*n* = 1,343, 44.8% before lockdown, *n* = 1,481, 49.4% during lockdown, *P* < 0.001). This was a significant increase in households with multiple adults, both with and without children. However, once adjusted for age, gender and ethnicity, the change seen in households with multiple adults and no children was no longer significant (*P* > 0.05) (Table 3).

**TABLE 3:** Frequently*^a^* ate meals at the dinner table before and during lockdown.

	Before lockdown, *n* (%)	During lockdown, *n* (%)	*p*-value^b^	Adjusted^c^ odds Ratio (95%CI)	Odds ratio *p*-value^d^
Single adult (*N* = 292)	139 (47.6)	148 (50.7)	0.508	0.93 (0.71, 1.22)	0.609
Households with 2 + adults (*N* = 1,601)	722 (45.1)	782 (48.8)	0.037	1.02 (0.86, 1.22)	0.781
Households with 2 + adults and 1 + child (*N* = 1,059)	465 (43.9)	531 (50.1)	0.005	1.00 (REF)	–
Households with 1 adult and 1 + child (*N* = 48)	17 (35.4)	20 (41.7)	0.675	1.4 (0.78, 2.51)	0.246

*^a^Frequently classified as responses “Frequently,” “Very frequently,” or “All of the time.”*

*^b^Fisher’s exact test.*

*^c^Adjusted for age group, gender, and ethnicity. Ref = households with 2 + adults and 1 + child.*

*^d^Wald’s test.*

There was an increase in the proportion of the survey respondents who stated that they frequently ate meals in front of a screen during lockdown (*n* = 972, 32.4% before lockdown, *n* = 1,095, 36.5% during lockdown, *P* < 0.001). This remained significant in households with two or more adults after adjusting for covariates, but not single person households (Table 4).

**TABLE 4:** Frequently*^a^* ate meals in front of a screen before and during lockdown.

	Before lockdown, *n* (%)	During lockdown, *n* (%)	*p*-value^b^	Adjusted^c^ odds ratio (95%CI)	Odds ratio *p*-value^d^
Single adult (*N* = 292)	78 (26.7)	86 (29.5)	0.519	1.7 (1.27, 2.28)	<0.001
Households with 2 + adults (*N* = 1,601)	440 (27.5)	529 (33.0)	<0.001	1.48 (1.24, 1.77)	<0.001
Households with 2 + adults and 1 + child (*N* = 1,059)	437 (41.3)	465 (43.9)	0.235	1.00 (REF)	–
Households with 1 adult and 1 + child (*N* = 48)	17 (35.4)	15 (31.3)	0.829	1.69 (0.9, 1.46)	0.1

*^a^Frequently classified as responses “Frequently,” “Very frequently,” or “All of the time.”*

*^b^Fisher’s exact test.*

*^c^Adjusted for household type, age group, gender, and ethnicity.*

*^d^Wald’s test.*

There was a substantial decrease in the proportion of respondents who ate out frequently at a restaurant or café during the lockdown (*n* = 878, 29.3% before lockdown, *n* = 5, 0.2% during lockdown, *P* < 0.001). This was a significant decrease across all household composition subgroups (*P* < 0.05). There was a decrease in the proportion of respondents who frequently used delivery or takeaway services for main meals during lockdown among all respondents (*n* = 650, 21.7% before lockdown, *n* = 22, 0.7% during lockdown, *P* < 0.001). This was a significant decrease across all household composition subgroups (*P* < 0.001) (Table 5).

**TABLE 5:** Frequently^a^ used delivery or takeaway services before and during lockdown.

	Before lockdown, *n* (%)	During lockdown, *n* (%)	*p*-value^b^
Single adult (*N* = 292)	44 (15.1)	6 (2.1)	<0.001
Households with 2 + adults (*N* = 1,601)	351 (21.9)	12 (0.7)	<0.001
Households with 2 + adults and 1 + child (*N* = 1,059)	240 (15.0)	4 (0.4)	<0.001
Households with 1 adult and 1 + child (*N* = 48)	15 (31.3)	0 (0.0)	<0.001

*^a^Frequently classified as responses “Frequently,” “Very frequently,” or “Every time I ate a warm meal.”*

*^b^Fisher’s exact test.*

There was an overall increase in the number of respondents who stated they used meal kit services for main meals during lockdown (*n* = 293, 9.8% before lockdown, *n* = 359, 12.0% during lockdown, *P* = 0.007). Single person households were half as likely to use meal kit services as the household composition group with 2 or more adults and children (AOR: 0.54, *P* = 0.014) (Table 6).

**TABLE 6:** Frequently*^a^* used meal box or ingredient kit services before and during lockdown.

	Before lockdown, *n* (%)	During lockdown, *n* (%)	*p*-value^b^	Adjusted^c^ odds ratio (95%CI)	Odds ratio *p*-value^d^
Single adult (*N* = 292)	19 (6.5)	20 (6.8)	1.0	0.54 (0.33, 0.88)	0.014
Households with 2 + adults (*N* = 1,601)	125 (7.8)	159 (9.9)	0.040	0.79 (0.61, 1.02)	0.071
Households with 2 + adults and 1 + child (*N* = 1,059)	144 (13.6)	168 (15.9)	0.158	1.00 (REF)	–
Households with 1 adult and 1 + child (*N* = 48)	5 (10.4)	12 (25.0)	0.107	1.76 (0.89, 3.48)	0.105

*^a^Frequently classified as responses “Frequently,” “Very frequently,” or “Every time I ate a warm meal.”*

*^b^Fisher’s exact test.*

*^c^Adjusted for household type, age group, gender, and ethnicity.*

*^d^Wald’s test.*

Survey questions asked about respondents’ experience of e-dining during the lockdown period. Respondents were asked to respond to the prompt *‘since your lockdown began, how often have you organized or participated in dinner with someone via online video chat?’.* Most of the survey respondents (*n* = 2,639, 88%) responded that they “(Almost) never” organized or participated in dinner with someone via online video chat (e-dining) during lockdown. One in ten respondents (*n* = 317, 10.6%) had engaged in e-dining once a week or less during lockdown, and 44 (1.4%) e-dined more often than once a week. There were no significant differences between the household composition groups in the frequency of e-dining (*P* = 0.604). Single-parent households were excluded from the analysis of attitudes toward e-dining, as the sample size of respondents in that group was too small to draw conclusions from (*n* < 5).

### Psychological Distress During Lockdown

**FIGURE 1:**
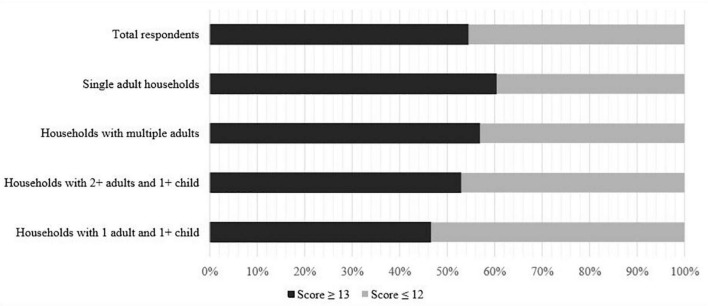
Respondents experience of psychological distress during lockdown.

Figure 1 presents the proportion of respondents experiencing psychological distress during lockdown (a score of 13 or more on the Kessler-6) by household composition group. The total scores amongst all respondents ranged from 6 (the lowest possible score) to 30 (the highest possible score). Just over half (*n* = 1,636, 54.5%) of all respondents self-reported a score of 13 or higher and among those the results varied between household composition groups from *n* = 136, 46.6% in the single person household group to *n* = 29, 60.4% in the single-parent households.

Respondents who experienced psychological distress during lockdown were 1.47 (95% CI 1.26–1.72, *P* < 0.001) times more likely to consider mealtimes an important part of their day than those who scored 12 or fewer. They were also 1.25 (95% CI 1.07–1.45, *P* = 0.004) times more likely to frequently eat dinner at the table and were 1.19 (95% CI 1.02–1.40, *P* = 0.029) times more likely to eat dinner in front of a screen or television. There were no significant differences by levels of psychological distress for those who frequently used meal kit services (AOR: 0.97,95% CI 0.77–1.22, *P* = 0.794) or engaged in e-dining (AOR: 1.03,95% CI 0.81–1.30, *P* = 0.811).

Table 7 details associations between psychological distress and mealtime behaviors by household type. In households with multiple adults but no children, those with psychological distress were 1.60 times (95% CI 1.28–1.99, *p* < 0.001) more likely to consider meals an important part of their day than those who scored a 12 or below. In households with two or more adults and at least one child, those with psychological distress were significantly more likely to consider mealtimes an important part of the day (AOR: 1.30, 95% CI 1.02–1.67, *p* = 0.037), eat dinner at the dinner table (AOR: 1.4, 95% CI 1.10–1.8, *P* = 0.007), and eat dinner in front of the television (AOR: 1.45, 95% CI 1.13–1.86, *P* = 0.004) compared to those who scored 12 or below. In households with two or more adults and at least one child, the group who scored 13 or above were 0.65 (95% CI 0.44–0.96, *P* = 0.029) times less likely to engage in e-dining than those who scored 12 or below. In households with one adult and at least one child, those with psychological distress were 5.95 (95% CI 1.47–24.14, *P* = 0.012) times more likely to eat dinner at the dinner table frequently than those who scored 12 or below (Table 7).

**TABLE 7:** The impact of psychological distress during lockdown on mealtime behaviors.

	Single person households	Households with 2 + adults and no children	Households with 2 + adults and 1 + child	Households with 1 adult and 1 + child
**Frequently^c^ considered mealtimes to be an important part of the day**				
Crude odds ratio (95%CI)	1.50 (0.90, 2.50)	1.58 (1.29, 1.94)	1.29 (1.01, 1.65)	2.29 (0.66, 7.95)
Adjusted^a^ odds ratio (95%CI)	1.62 (0.92, 2.87)	1.60 (1.28, 1.99)	1.30 (1.02, 1.67)	1.92 (0.51, 7.27)
*P*-value^b^	0.095	<0.001	0.037	0.338
**Frequently^c^ ate dinner at the dinner table**				
Crude odds ratio (95%CI)	0.94 (0.60, 1.49)	1.10 (0.91, 1.35)	1.35 (1.06, 1.72)	4.50 (1.31, 15.32)
Adjusted^a^ odds ratio (95%CI)	0.86 (0.52, 1.43)	1.17 (0.95, 1.45)	1.4 (1.10, 1.80)	5.95 (1.47, 24.14)
*P*-value^b^	0.563	0.138	0.007	0.012
**Frequently^c^ ate dinner in front of the television**				
Crude odds ratio (95%CI)	0.94 (0.57, 1.56)	1.05 (0.85, 1.3)	1.38 (1.08, 1.76)	0.68 (0.19, 2.43)
Adjusted^a^ odds ratio (95%CI)	0.92 (0.53, 1.59)	1.06 (0.85, 1.33)	1.45 (1.13, 1.86)	0.65 (0.16, 2.7)
*P*-value^b^	0.758	0.592	0.004	0.554
**Frequently^c^ used meal kit or ingredient box services**				
Crude odds ratio (95%CI)	1.16 (0.47, 2.87)	1.28 (0.91, 1.80)	0.87 (0.63, 1.21)	1.43 (0.36, 5.63)
Adjusted^a^ odds ratio (95%CI)	0.75 (0.27, 2.06)	1.13 (0.79, 1.61)	0.84 (0.60, 1.18)	1.26 (0.29, 5.37)
*P*-value^b^	0.577	0.517	0.324	0.758
**Engaged in e-dining during the lockdown^d^**				
Crude odds ratio (95%CI)	2.27 (1.17, 4.4)	1.56 (1.14, 2.14)	0.70 (0.48, 1.03)	-^e^
Adjusted^a^ odds ratio (95%CI)	1.53 (0.73, 3.17)	1.23 (0.88, 1.73)	0.65 (0.44, 0.96)	-^e^
*P*-value^b^	0.257	0.226	0.029	-^e^

*^a^Adjusted for age, gender, and ethnicity.*

*^b^Wald’s test.*

*^c^Frequently included the responses “frequently,” “very frequently,” and “all of the time.”*

*^d^Included all respondents who reported engaging in e-dining at least once during the lockdown.*

*^e^Households with one adult and one or more child excluded from analysis of e-dining due to low response numbers.*

## Discussion

The findings of this study indicate that the mealtime behaviors of cooking meals at home and eating meals at the dinner table increased during the first COVID-19 lockdown in Aotearoa New Zealand for all groups and especially for those in households with children and for participants who experienced psychological distress. Eating out and getting takeaways massively decreased for all participants over this period.

### The Rise in Meal Kits and Decrease in Takeaways

The findings of this study show an overall increase in the perceived importance of mealtimes and an increase in meals cooked and prepared at home. Subsequently the use of meal kit services also increased during the lockdown period. The use of meal kits or ingredient box services may indicate that people were cooking more meals at home, as meal kit services were delivered to the household address with recipes and all necessary ingredients included. A study by Romeo-Arroyo et al. (16) explains that during confinement, the amount that a person cooks is dependent on their perception of cooking as either a pleasure or a duty. When cooking for oneself, there is less enjoyment in the process of cooking a meal, whereas cooking for or with others can be a form of entertainment and strengthens social bonds (16, 31, 32). Carroll et al. (32) discuss that during the COVID-19 lockdown in Canada, parents used cooking to bond with children, keep them busy, and reduce screen time. Meal kit use was greatest in households with children compared to households without children, which may be due to the added time pressures on adults who need to balance work and childcare responsibilities alongside the ease of children being engaged in the process of preparing meals.

The Aotearoa New Zealand COVID Health Survey found that at the beginning of the first lockdown in April 2020, 26% of respondents reported feeling stressed about leaving home, thus making grocery shopping more challenging (33). An alternative option would be getting one’s groceries delivered by the store. However, in Aotearoa New Zealand there were long wait times for grocery deliveries as many people wanted to use the service and grocery stores were prioritizing populations with the most need, such as the elderly and disabled (34). Meal boxes were an alternative option for people who were unable to get their groceries delivered and were hesitant to visit the grocery store during lockdown. A possible explanation for why meal kit use was lowest in single-person households may be that most ingredient boxes are designed for at least two people. Purchasing a meal kit just for one person may not be financially viable and/or result in greater food waste (26). Meal kits would benefit from including information about how to modify recipes to reduce energy intake or suggest alterations in portion size for those with lower energy requirements or one person (35).

Before lockdown, 29.3% (*n* = 878) of respondents stated that they ate out frequently, and 21.7% (*n* = 650) reported frequently getting takeaways. These proportions decreased immensely during lockdown most likely because the lockdown restrictions in Aotearoa New Zealand meant that restaurants and cafes were not allowed to open at all during Level 4, although they were able to open for takeaway and contactless delivery during Level 3. So, it would not have been possible for respondents to frequently eat out. However, the significant decrease in takeaway and delivery services cannot totally be explained so easily, as 66.6% of the COVID Kai Survey responses were collected during Level 3 restrictions (24), meaning that takeaway services were available for most people when they completed the survey. Studies about lockdowns in other countries also found that people are less likely to eat takeaway food during lockdown (10, 32), with concerns about price, safety, or greater motivation to eat healthy foods as potential reasons why takeaway use decreased for our survey respondents during the lockdown. However, anecdotal evidence shows that many businesses were swamped with customers once they opened for takeaways at the start of Level 3 (36). Therefore, the low numbers of people who reported eating takeaways frequently may be an outcome of the survey population demographics rather than an accurate representation of Aotearoa New Zealanders eating behaviors during lockdown.

### Increased Mealtimes at the Table but Also Still Eating in Front of Screens

This study found an increase in the frequency of households eating at the dinner table during lockdown, and an increase in the perceived importance of mealtimes. This may be because during lockdown many people felt that they were missing a sense of routine (37) and mealtimes provide a sense of routine that was otherwise missing without school/work. Engaging in a routine, such as eating dinner at the table, has also been shown to be a coping strategy for people in times of stress and give people a sense of task-accomplishment (38, 39). Findings from the USA indicate that eating meals regularly at the dinner table gave respondents a sense of normalcy and acted as an important grounding time during the uncertainty of COVID-19 (40). In this study, households with two or more adults and children had the largest increase in eating at the table more frequently. Eating meals at the table has been shown to benefit adults and children because it is related to making healthier food choices, increasing family connection, improving mental health outcomes, and discouraging engagement in high-risk behaviors such as alcohol abuse (21). This may explain why respondents who experienced psychological distress and who resided in households with children experienced the largest increase in eating at the table more frequently.

Eating in front of a screen is not considered healthy eating behavior as this is associated with increased dietary intake and the inability to notice when you are full (41–43). For households of more than one person, screen use during mealtimes may be considered harmful as it creates a barrier to connect with others (44). Single person households and households with multiple adults and no children were significantly more likely to eat meals in front of the television than households with 2 or more adults and children, whereas households with children were more likely to eat dinner at the table. It has been found that during COVID-19 lockdowns globally, overall screen time increased so it makes sense that respondents reported spending more time eating in front of screens (45–47).

E-dining grew in popularity during lockdown whereby people in different households could eat together while on video conferencing software. This acted as a mode of social interaction during a time of physical distancing and isolation. There were anecdotal stories that some people began to hold virtual dinner parties to maintain some form of food-related socialization (48). However, our results found that only 12% of respondents reported e-dining regularly. Although e-dining is a mode of social interaction, it may only fulfill some of the benefits of face-to-face meal sharing. Some of the protective elements transferable to e-dining are; socialization, support, a strengthened sense of community, and a sense of control and normalcy in uncertain times (40). What is missing from e-dining is the actual sharing of food and resources alongside the opportunity to meet new members of the community and develop connections. For e-dining, one generally needs to be invited to an online meeting room, so those participating will most likely already know each other. There are also some general barriers to e-dining that may answer why so few people engaged in the behavior. For example, to engage in online meal sharing, a person must have access to a computer with a microphone and a camera and a reliable internet connection. These barriers mean that financially disadvantaged people may have limited access to e-dining even though they are the group that traditionally has benefitted most from meal-sharing practices during a crisis (49–51). This survey was administered during the first major lockdown in Aotearoa New Zealand. People were still grappling with it and had not yet relaxed into a COVID-19 world. There is the potential that if this survey were repeated during the subsequent lockdowns, e-dining would have been more common.

### Single-Parent Households Had the Highest Stress and This Was Associated With Beneficial Mealtime Behaviors

During crises, psychological distress can arise from financial insecurity, food insecurity, general uncertainty, isolation, exacerbation of previous mental health conditions, and/or an insecure home life. All of these factors were relevant during the first Aotearoa New Zealand lockdown. Our results also showed a decrease in people engaged in full-time employment among all household groups and an increase in people who did not work during lockdown. A scoping review of the impact of eating behaviors during recent crisis indicates that precarious employment is a critical factor in stress levels and negatively impacts eating behaviors (52). This is important to consider for COVID-19, as many industries shut down, and at its worst in September 2020, approx. 151,000 people in Aotearoa New Zealand were unemployed. This represents a 32.5% increase since the end of the previous quarter in June 2020, a rise attributed to the impact of COVID-19 (53).

Single parent households had the highest levels of financial struggle during lockdown, yet they also reported the lowest levels of income lost, most likely because a high proportion of single parent households receive government welfare payments (54). In Aotearoa New Zealand single-parent households are on average financially worse off than other types of households, and in 2020, 18% of single-parent households did not have enough money to meet their everyday needs (55). Our results showed that among all household types single parent households reported the highest levels of self-reported psychological distress. This may be explained because distress is highest in situations where people are financially insecure and also because of the stress of balancing the demands of working from home while also being solely responsible for child care and home schooling (56). Our findings of increased psychological distress among single parent households align with international research where parents have reported increased stress during the COVID-19 lockdown (56–58). Reasons for this are reportedly to relate to school closures and the difficulty faced working from home (57), alongside financial hardship and concern over children’s mental and physical health (58, 59).

Psychological distress experienced in all households with children during the lockdown appears to have been accompanied by increases in beneficial mealtime behaviors, such as eating at the dinner table. This is consistent with previous research conducted in times of crisis showing that parents will utilize the skills they have available to them; specifically installing routines and it is possible parents capitalized on the lockdown to spend quality time together as a household (19). Findings of children’s perceptions of lockdown in Aotearoa New Zealand reveal that children loved the additional time lockdown afforded them to spend with parents and household members (60). It is also possible that these increases in beneficial mealtime behaviors occurred as a result of decreased meals consumed outside of the home, in restaurants, cafes and takeaways eaten in the car or in a public place, but further research is necessary to explore these connections and confirm directionality.

### Strengths and Limitations

To the authors’ best knowledge this research is the first to explore changes to mealtime behaviors during the COVID-19 lockdown in Aotearoa New Zealand and the first to report a significant increase in beneficial mealtime behaviors, such as eating meal at the dinner table and decreased eating out among single parent households and among those experiencing psychological distress. The timeliness in which this study was completed is a strength of the research. The COVID-19 pandemic is rapidly changing and remains a contemporary influence on people’s ability to acquire food and will likely remain an influence on stress and mealtime behaviors for some time to come (61, 62).

A further strength of the study is that the data was collected whilst Level 3 and 4 lockdowns were still in place. Although the data collection methods were retrospective and self-reported, respondents answered questions while still in the period of interest. Consequently, the results likely reflect the lockdown experience as it was fresh in the respondent’s minds. Overall, the study had a high participation rate given it was conducted during a period of uncertainty and restricted movement. In comparison to other countries that participated in the Corona Cooking Survey project, the Aotearoa New Zealand branch had significantly higher response numbers per head of population (23). The online format of the COVID Kai Survey meant respondents did not have to take any risks in terms of safety regarding COVID-19 in order to participate.

Funding was obtained that allowed for a $1 koha to be donated to the Aotearoa New Zealand Food Bank for every response collected. This was a strength of the research as it gave New Zealanders an additional reason to participate in the study, as well as an opportunity to do something beneficial in a time when many people felt helpless (63). It is also considered good practice for research initiatives to give back to the community from which they collect data rather than simply taking from it.

One of the main limitations of this study is that the respondents were not representative of the Aotearoa New Zealand population. Respondents were primarily well-educated, New Zealand European people who identified as women. There was a very low representation of Pacific people and gender diverse people. The use of an online survey format promoted through social media favored people with privilege. Online data collection is not suitable for collecting information about Māori and Pacific people due to cultural barriers (64). To effectively engage with Māori and Pacific people, it is necessary for researchers to take the time to build authentic relationships through face-to-face engagement. Unfortunately, due to the physically distanced nature of the COVID-19 lockdown this was not possible. Zoom interviews could have been a potential way to establish these relationships in a COVID-19 friendly way (65). The COVID Kai research team worked with cultural organizations to develop advertisements for the survey in Te Reo Māori and a variety of Pacific languages and promoted the survey through their networks. However, this was ultimately unsuccessful at recruiting sufficient numbers to be representative of the national population.

Even for English speakers, the survey required a high literacy level to complete and had a significant participant burden, taking around 30 min. If this study were repeated, it would be helpful to amend the questions to be more appropriate for the Aotearoa New Zealand population and consider other modes of data collection such as targeted phone, text message or Zoom interviews. Offering a larger koha directly to the participant may also incentivize more people to contribute. The household group distributions were also not representative of Aotearoa New Zealand; the single-parent household group had a low response number, even though one-third of families in Aotearoa New Zealand are headed by a single parent (66). This may be because sole parents and their children moved in with their extended families during lockdown, or they were just too stressed or busy to be able to dedicate sufficient time to participate.

Another limitation of the findings is that the Likert scales as response categories had no clear guidance as to what each point on the scale meant. The responses were likely interpreted differently by different individuals (67). For example, what one respondent would have considered “rarely” engaging in a behavior, another may have considered “sometimes.” To simplify the results and avoid bias associated with misinterpretation of the scales, the results were collated into binary categories, “less than frequently” and “frequently or more” in the analyses presented but this would have resulted in lost detail.

Due to the retrospective and self-reported nature of the “before-pandemic” questions asked, there is potential bias in the data collected. Self-reported data, particularly about food and eating behaviors, has the potential for bias due to selective recall and social desirability impacting what a respondent chooses to report (68). Often unhealthy and less socially desirable behaviors are not as easily recalled and thus are underreported (68). However, people are more likely to be honest in surveys when they are completed independently, as opposed to through face-to-face interviewing (69). Selective recall can also be due to respondents re-evaluating their own behaviors over time and choosing not to disclose some details (68). This is particularly common in nutrition studies as food choice is a sensitive topic and people will often modify their responses in order to come across as healthier (68, 70). This is also often the case in research conducted on parenting where answers may be edited for social desirability (71). This issue was minimized somewhat by the short recall period and by assuring respondents that all data collected was anonymous.

Additionally, one of the main measures in the study was psychological distress. However, no potential positive psychological aspects of lockdown were measured. A large study of Māori conducted at the same time as the COVID Kai Survey found 19.5% of responders reported positive whānau (family) outcomes and 17.1% reported positive psychological outcomes due to the COVID-19 lockdown, with nearly 14% reporting that lockdown gave people an opportunity to stop and reflect on their lives (72). Children too reported that they liked many aspects of lockdown including the slower pace of life and the increased time spent with family doing simple everyday activities such as going for bike rides in their neighborhood, pajama days and playing games together (60).

### Implications for Future Research

Our recommendations for future research are to undertake research with a sample that is more representative of the total population in Aotearoa New Zealand. Māori made up 10.5% of respondents, and Pacific people made up only 2.7% (Table 4). These proportions are low compared to the demographic population of Aotearoa New Zealand, where 16.7% of the population is Māori, and 8.3% of the population is Pacific (73). Our responsibility in Aotearoa New Zealand is to uphold Te Tiriti O Waitaingi and ensure that Māori perspectives are represented in all research areas, and that evidence-based policies reflect the needs of Māori to minimize inequities between Māori and non-Māori (74). Future research on eating behaviors during lockdown that utilizes a Kaupapa Māori approach is needed. Having a comprehensive understanding of the impact of the pandemic on all people in Aotearoa New Zealand is imperative to inform more equitable policy decisions.

This study investigated the extent to which mealtime behaviors changed during lockdown but could not thoroughly investigate why these behaviors changed. The literature on mealtime planning in low-income families is lacking. Family dynamics and food insecurity may potentially have an impact and more research in that area is needed. The scope of a quantitative survey study design meant that there was minimal context available regarding the participant’s experiences of lockdown or why they felt their behaviors changed. A qualitative research approach would address this gap and could be achieved through interviews and/or analyzing social media content. The Zoom focus group method used by Hammons and Robart (40) and described in detail by Pocock et al. (65) would be a good option for conducting qualitative research in the event of another lockdown. Qualitative studies to explore people’s experiences and perceptions of mealtime behaviors and stress during lockdown would be particularly beneficial to understand more about why our findings revealed both an increase in stress and an increase in beneficial mealtime behaviors. It would also be interesting to see if these behaviors adopted during the first COVID-19 lockdown were maintained once lockdown restrictions eased, or if old habits and routines were reinstated.

Increasing the availability of funding for qualitative research would enable researchers and policymakers to understand the experiences of Aotearoa New Zealanders in lockdown more thoroughly.

## Conclusion

This study investigated changes in mealtime behaviors during the first 2020 COVID-19 lockdown in Aotearoa, New Zealand. Data from the COVID Kai Survey indicated that cooking meals at home, eating meals at the dinner table, and considering mealtimes to be an important part of the day, all increased during lockdown. Eating out or getting takeaways, decreased over this period. Across most household types, people who were psychologically distressed during lockdown were more likely to consider mealtimes as an important part of the day. Those who reported psychological distress and resided in households with children were more likely to eat dinner at the dinner table. Single-parent households reported the highest rates of financial hardship, and psychological distress. This study advances current understanding of mealtime behaviors during crises and adds to the growing body of literature regarding the everyday impacts of COVID-19. Further research is required to fully understand the experience of psychological distress on mealtime behaviors with a representative sample of people residing in Aotearoa New Zealand. Qualitative studies that expand on the reasons behind behavior change are needed.

## Data Availability Statement

The datasets presented in this article are not readily available because we did not receive ethical approval to share raw, anonymized data with others. Requests to access the datasets should be directed to corresponding author.

## Ethics Statement

The Corona Cooking Survey was granted ethical approval by the Ethics Advisory Committee on Social and Human Science at the University of Antwerp on April 16th 2020 (ref: SHW_20_46). The Aotearoa New Zealand arm of the study was granted ethical approval by the University of Auckland Human Respondents Ethics Committee on 24 April 2020 for 3 years (ref: 024607). The patients/participants provided their written informed consent to participate in this study.

## Author Contributions

VE, LH, RR, LTM, LT, PD, IC, CD, and SG: conceptualization, data curation, investigation, and methodology. LH, VE, and SG: formal analysis and writing—original draft. VE, RR, LTM, LT, PD, IC, CD, and SG: funding acquisition. RR, LTM, LT, PD, IC, and CD: writing—review and editing. All authors have read and agreed to the published version of the manuscript.

## Conflict of Interest

The authors declare that the research was conducted in the absence of any commercial or financial relationships that could be construed as a potential conflict of interest.

## Publisher’s Note

All claims expressed in this article are solely those of the authors and do not necessarily represent those of their affiliated organizations, or those of the publisher, the editors and the reviewers. Any product that may be evaluated in this article, or claim that may be made by its manufacturer, is not guaranteed or endorsed by the publisher.
